# Dynamic Spatiotemporal Expression Changes in Connexins of the Developing Primate’s Cochlea

**DOI:** 10.3390/genes12071082

**Published:** 2021-07-16

**Authors:** Makoto Hosoya, Masato Fujioka, Ayako Y. Murayama, Kaoru Ogawa, Hideyuki Okano, Hiroyuki Ozawa

**Affiliations:** 1 Department of Otorhinolaryngology, Head and Neck Surgery, Keio University School of Medicine, 35 Shinanomachi Shinjuku-ku, Tokyo 160-8582, Japan; mhosoya1985@gmail.com (M.H.); ogawak@keio.jp (K.O.); ozakkyy123@gmail.com (H.O.); 2 Department of Physiology, Keio University School of Medicine, 35 Shinanomachi Shinjuku-ku, Tokyo 160-8582, Japan; murasann0717@yahoo.co.jp (A.Y.M.); hidokano@a2.keio.jp (H.O.); 3 Laboratory for Marmoset Neural Architecture, RIKEN Center for Brain Science, Wako 351-0198, Japan

**Keywords:** cochlea, inner ear, common marmoset, primate, connexin

## Abstract

Connexins are gap junction components that are essential for acquiring normal hearing ability. Up to 50% of congenital, autosomal-recessive, non-syndromic deafness can be attributed to variants in *GJB2*, the gene that encodes connexin 26. Gene therapies modifying the expression of connexins are a feasible treatment option for some patients with genetic hearing losses. However, the expression patterns of these proteins in the human fetus are not fully understood due to ethical concerns. Recently, the common marmoset was used as a primate animal model for the human fetus. In this study, we examined the expression patterns of connexin 26 and connexin 30 in the developing cochlea of this primate. Primate-specific spatiotemporal expression changes were revealed, which suggest the existence of primate-specific control of connexin expression patterns and specific functions of these gap junction proteins. Moreover, our results indicate that treatments for connexin-related hearing loss established in rodent models may not be appropriate for human patients, underscoring the importance of testing these treatments in primate models before applying them in human clinical trials.

## 1. Introduction

Hearing loss is the most frequent congenital sensory impairment, with deafness occurring in approximately 1–2 per 10,000 newborns and any hearing loss occurring in 1–2 per 1000 [1,2,3]. The cause of this hearing loss is monogenic in approximately half of cases [4]. Mutations in genes causing congenital hearing loss can affect auditory ability through a variety of mechanisms, including effects on cochlear sensory epithelium development, neuronal conduction, and hair cell mechanosensory activity. Connexins have received considerable attention in the field of hereditary hearing loss because up to 50% of cases of congenital, autosomal-recessive, non-syndromic deafness can be attributed to variants in the gene *GJB2*, which encodes connexin 26 (CX26) [5,6,7,8,9].

Mutations in *GJB2* cause variable levels of congenital hearing loss, with most patients having nonprogressive hearing loss first observed at birth without malformations in their cochleae [6,10]. Transgenic mouse models with reduced levels of cochlear Cx26 expression demonstrate increased hearing thresholds and a decreased endocochlear potential, with loss of hair cells [11,12,13,14] and active cochlear amplification [15]. Therefore, CX26 mutations are thought to disturb the normal development and maintenance of the sensory epithelium of the cochlea. *GJB6*, which encodes connexin 30 (CX30), is another well-known causative gene for hearing loss. Rodent animal models have revealed that mutations in *Gjb6* also cause congenital hearing loss with sensory epithelium degeneration and a lack of the endocochlear potential [16].

These two connexins have close clinical and biological relationships. CX26 and CX30 have an overlapping distribution of expression in the cochlea [17,18,19]. Additionally, in clinical practice, heterozygous mutations in *GJB2* and *GJB6* are often observed [20]. It is also known that CX26 and CX30 can form functional heterotypic/heteromeric gap junction channels, with digenic Cx26 and Cx30 mutations impairing heterozygous coupling of Cx26 and Cx30 in the lateral cochlear wall, which leads to reductions in the endocochlear potential followed by hearing loss [21]. In addition, a relationship between the expression level of *GJB6* and the severity of hearing loss caused by *GJB2* mutations has been suggested [22].

Recently, several treatment strategies for hereditary hearing loss were developed, including the administration of small molecular compounds [23,24], cell therapy [25,26], and gene therapy [27,28,29,30,31]. For human patients with *GJB2/GJB6* mutations, compensation with normal connexin proteins through viral transduction with adeno-associated viral vectors is thought to be a possible treatment [32,33,34]. However, for efficient clinical results, transfer of these viral vectors is needed in utero or at birth. Unfortunately, essential knowledge about these genes, including their expression patterns, is not fully understood in humans due to technical and ethical concerns.

Recently, a primate model animal, the common marmoset, was established as a suitable nonhuman alternative for studies of cochlear development [35] and hereditary hearing loss [36,37,38]. Combining knowledge obtained from this model primate with information about the human fetus will help us to understand inter-species differences. Here, we investigated the expression patterns of CX26 and CX30 in the developing primate cochlea.

## 2. Materials and Methods

### 2.1. Specimens

Cadaverous, fixed, temporal bone samples of common marmosets at E96 (*n* = 5), E101 (*n* = 4), E115 (*n* = 4), and P0 (*n* = 5) were kindly provided by the Ayako Murayama and the Central Institute for Experimental Animals (CIEA).

The animal experiments were approved by the Animal Experiment Committee of Keio University (number: 11,006 and 08020) and RIKEN (H30-2-214[3]) and were conducted according to the guidelines of the National Institutes of Health and the Ministry of Education, Culture, Sports, Science, and Technology of Japan.

### 2.2. Tissue Preparation

The temporal bone region of each common marmoset embryo was dissected and fixed with 4% paraformaldehyde in PBS for 24 h immediately after euthanasia. P0 specimens were decalcified in decalcifying solution B (Wako, Osaka, Japan) for one week and then embedded in Tissue-Tek O.C.T. compound for cross-sectioning. Seven micrometer sections were used for immunohistochemical analyses.

### 2.3. Immunohistochemistry

After a brief wash with PBS, the sections were heated (80 °C) in a 10-µM citrate buffer (pH 6) for 15 min. After another brief wash, the sections were pre-blocked for 1 h at room temperature in 10% normal serum in PBS, incubated with primary antibodies at 4 °C overnight, and then incubated with Alexa Fluor-conjugated secondary antibodies for 60 min at room temperature. Nuclei were counterstained with Hoechst 33,258.

### 2.4. Antibodies

The following primary antibodies were used: mouse anti-connexin 26 antibody (Cx-12H10; #13-8100, 1:200. Invitrogen, Carlsbad, CA, USA, #13-8100), rabbit anti-connexin 30 antibody (Z-PP9) (#71-2200, 1:500, Invitrogen, Carlsbad, CA, USA), mouse anti-myosin7a antibody (#138-1-s, 1:30, DSHB, Iowa City, IA, USA), rabbit anti-myosin7a (#25-6790, 1:200, Proteus Biosciences, Ramona, CA, USA), goat anti-SOX2 antibody (AF2018, 1:200; R&D Systems, Minneapolis, MN, USA), and goat anti-NKCC1 (SLC12A2) antibody (sc21545, 1:300; Santa Cruz Biotechnology, Santa Cruz, CA, USA).

The following secondary antibodies were used: donkey anti-rabbit IgG, Alexa Fluor Plus 488 (A32790, 1:500, Invitrogen, Waltham, MA, USA), donkey anti-rabbit IgG, Alexa Fluor Plus 555 (A32794, 1:500, Invitrogen, Waltham, MA, USA), donkey anti-mouse IgG, Alexa Fluor Plus 488 (A32766, 1:500, Invitrogen, Waltham, MA, USA), donkey anti-mouse IgG, Alexa Fluor Plus 555 (A32773, 1:500, Invitrogen, Waltham, MA, USA), and donkey anti-goat IgG, Alexa Fluor 647 (705-605-147, 1:500, Jackson Immuno-Research, West Grove, PA, USA).

## 3. Results

### 3.1. Expression of CX26

**Figure 1:**
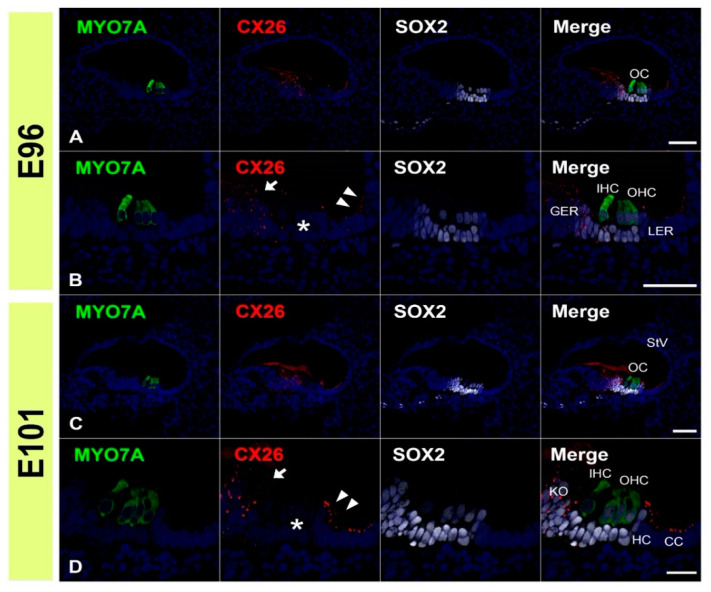
Changes in the expression patterns of CX26 at E96 and E101. (**A**,**B**) At E96, CX26 was expressed in the prosensory domain. While CX26 expression was observed in portions of the modiolus (arrow in (**B**)) and lateral (arrowhead in (**B**)) sides of the sensory epithelium, no expression was observed around the MYO7A-positive outer hair cells (asterisk in (**B**)). (**C**,**D**) At E101, CX26 expression was observed in Kölliker’s organ (arrow in (**D**)), immature Hensen’s cells (arrowhead in (**D**)), Claudius’ cells, and outer sulcus cells. No expression was observed in the organ of Corti (asterisk in (**D**)). SOX2 was used to label supporting cells. Nuclei were counterstained with Hoechst stain (blue). Scale bar: 50 µm in (**A**–**C**), 20 µm in (**D**). OC: organ of Corti, IHC, inner hair cells; OHC, outer hair cells, GER: greater epithelial ridge, LER: lesser epithelial ridge, StV: stria vascularis, KO: Kölliker’s organ, HC: Hensen’s cells, CC: Claudius’ cells, (**A**–**D**): basal turn.

**Figure 2:**
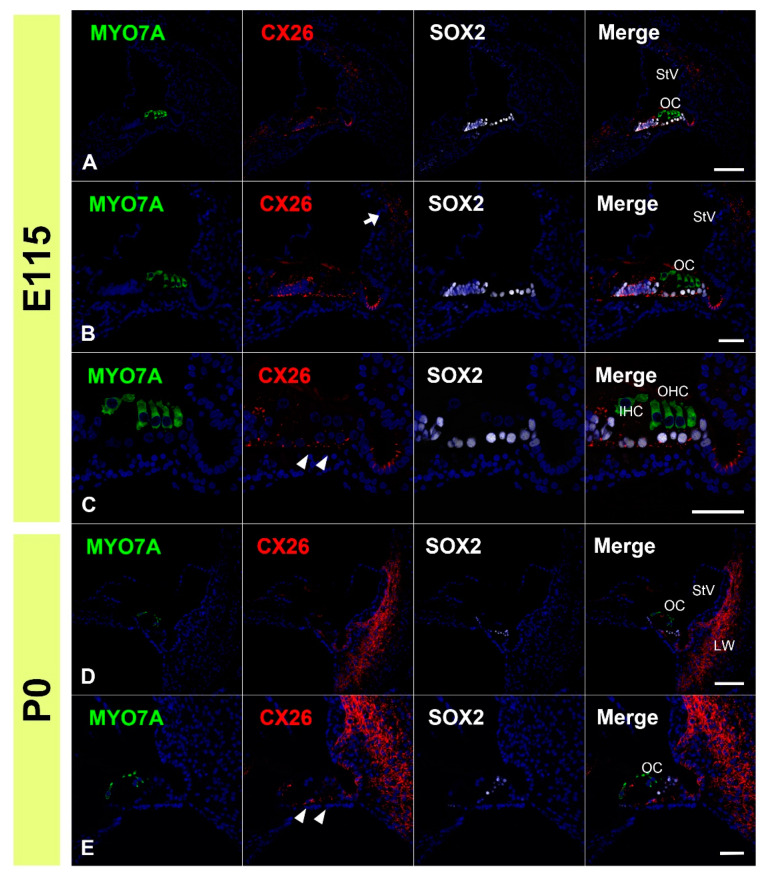
Changes in the expression patterns of CX26 at E115 and P0. (**A**–**C**) At E115, a broader expression of CX26 was detected in the sensory epithelium, including in the SOX2-positive supporting cells. However, no expression was observed in the MYO7A-positive hair cells. Weak expression was also observed in the spiral ligament fibrocytes next to the stria vascularis (arrow in (**B**)). At this stage, CX26 expression in the supporting cells was observed only near the basal-side membrane (arrowhead in (**C**)). (**D**,**E**) At P0, strong CX26 expression was observed in spiral ligament fibrocytes, while relatively weak expression was observed in the organ of Corti (arrowhead in (**E**)). Nuclei were counterstained with Hoechst stain (blue). Scale bar: 100 µm in (**A**,**D**), 50 µm in (**B**,**C**,**E**). OC: organ of Corti, IHC, inner hair cells; OHC, outer hair cells, StV: stria vascularis, LW: lateral wall fibrocytes, (**A**–**E**): basal turn.

First, we examined the expression patterns of CX26 in a common marmoset. At E96, CX26 was expressed in the prosensory domain (Figure 1A,B). While CX26 expression was observed in a part of the modiolus and lateral sides of the sensory epithelium, no expression was observed around the MYO7A-positive outer hair cells. At E101, CX26 expression was still observed in the sensory epithelium, though not in the organ of Corti (Figure 1C,D). At E115, CX26 expression was observed in the supporting cells of the organ of Corti (Figure 2A–C) but limited to the bottom side of the lateral membrane of these cells. No expression was observed in either type of hair cell. At this stage, weak expression was also observed in the spiral ligament fibrocytes next to the stria vascularis (Figure 2B). At P0, strong CX26 expression was observed in spiral ligament fibrocytes; however, expression in the sensory epithelium, including the organ of Corti, was diminished compared with E115. No expression was observed in either type of hair cell.

### 3.2. Expression of CX30

**Figure 3:**
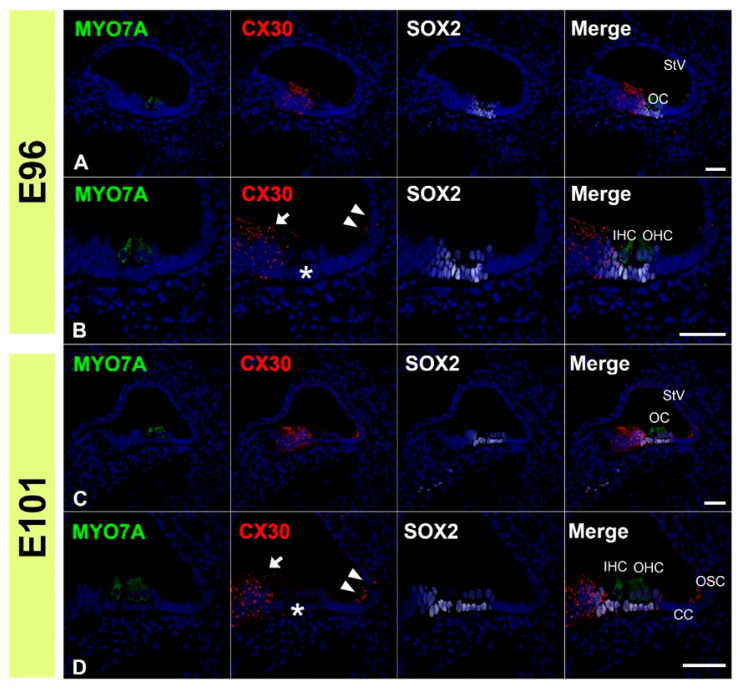
Changes in the expression patterns of CX30 at E96 and E101. (**A**,**B**) At E96, CX30 was expressed in the prosensory domain. While CX30 expression was observed in a part of the modiolus (arrow in (**B**)) and lateral (arrowheads in (**B**)) side of the sensory epithelium, no expression was observed around the MYO7A-positive outer hair cells (asterisk in (**B**)). (**C**,**D**) At E101, CX30 expression was observed in the Kölliker’s organ (arrow in (**D**)) and immature outer sulcus cells (arrowheads in (**D**)). No expression was observed in the organ of Corti (asterisk in (**D**)). SOX2 was used to label supporting cells. Nuclei were counterstained with Hoechst stain (blue). Scale bar: 50 µm in A, (**B**–**D**). OC: organ of Corti, IHC, inner hair cells; OHC, outer hair cells, StV: stria vascularis, CC: Claudius’ cells, OSC: outer sulcus cells. (**A**–**D**): basal turn.

**Figure 4:**
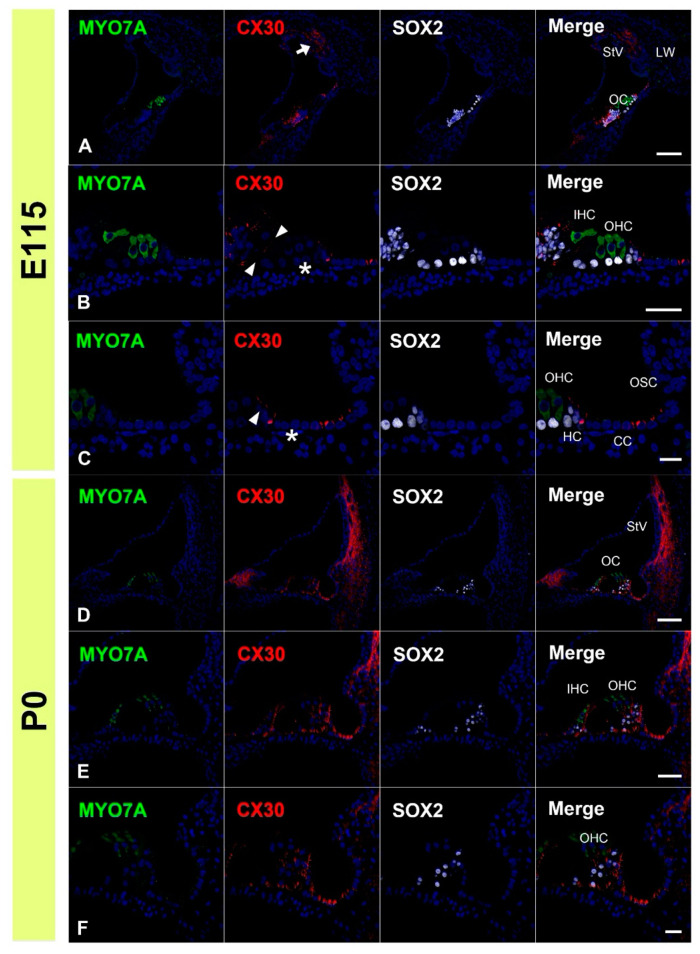
Changes in the expression patterns of CX30 at E115 and P0. (**A**–**C**) At E115, expression of CX30 was detected in a part of the sensory epithelium, but no expression was observed in the MYO7A-positive hair cells. Weak expression was observed in the spiral ligament fibrocytes next to the stria vascularis (arrow in (**A**)). CX30 expression was also observed at the modiolus-side membrane of inner pillar cells (arrowheads in (**B**)). At this stage, no CX30 expression was detected in Deiters’ cells (asterisk in (**B**)). In Hensen’s cells, however, expression of CX30 was detected (arrowhead in (**C**)). Notably, in Claudius’ cells, cells located on the modiolus side showed no expression of CX30 (asterisk in (**C**)), while expression was detected in cells on the spiral ligament side. (**D**–**F**) At P0, strong CX30 expression was observed in spiral ligament fibrocytes and the organ of Corti (arrowhead in (**E**)). SOX2 was used to label supporting cells. Nuclei were counterstained with Hoechst stain (blue). Scale bar: 100 µm in (**A**,**D**), 50 µm in B and E, 20 µm in (**C**,**F**). OC: organ of Corti, StV: stria vascularis, LW: lateral wall fibrocytes, IHC, inner hair cells; OHC, outer hair cells, CC: Claudius’ cells, HC: Hensen’s cells, OSC: outer sulcus cells. (**A**–**F**): basal turn.

Next, we examined the expression patterns of CX30 in the common marmoset. At E96, CX30 was expressed in the prosensory domain (Figure 3A,B). While CX30 expression was observed in portions of the greater epithelial ridge (GER) and lesser epithelial ridge (LER), no expression was observed around the outer hair cells. At E101, CX30 expression was observed in the Kölliker’s organ and immature outer sulcus cells (Figure 3C,D). No CX30 expression was observed in the organ of Corti. At E115, CX30 expression was detected in a part of the sensory epithelium (Figure 4A–C). While no expression was observed in either type of hair cell, CX30 expression was observed in several supporting cells of the organ of Corti, including the inner pillar cells and Hensen’s cells. Weak expression was also observed in the spiral ligament fibrocytes next to the stria vascularis (arrow in Figure 4A). At P0, strong CX30 expression was observed in the spiral ligament fibrocytes and in the organ of Corti (Figure 4D–F).

### 3.3. Comparing the Expression Patterns of CX26 and CX30 in the Sensory Epithelium

**Figure 5:**
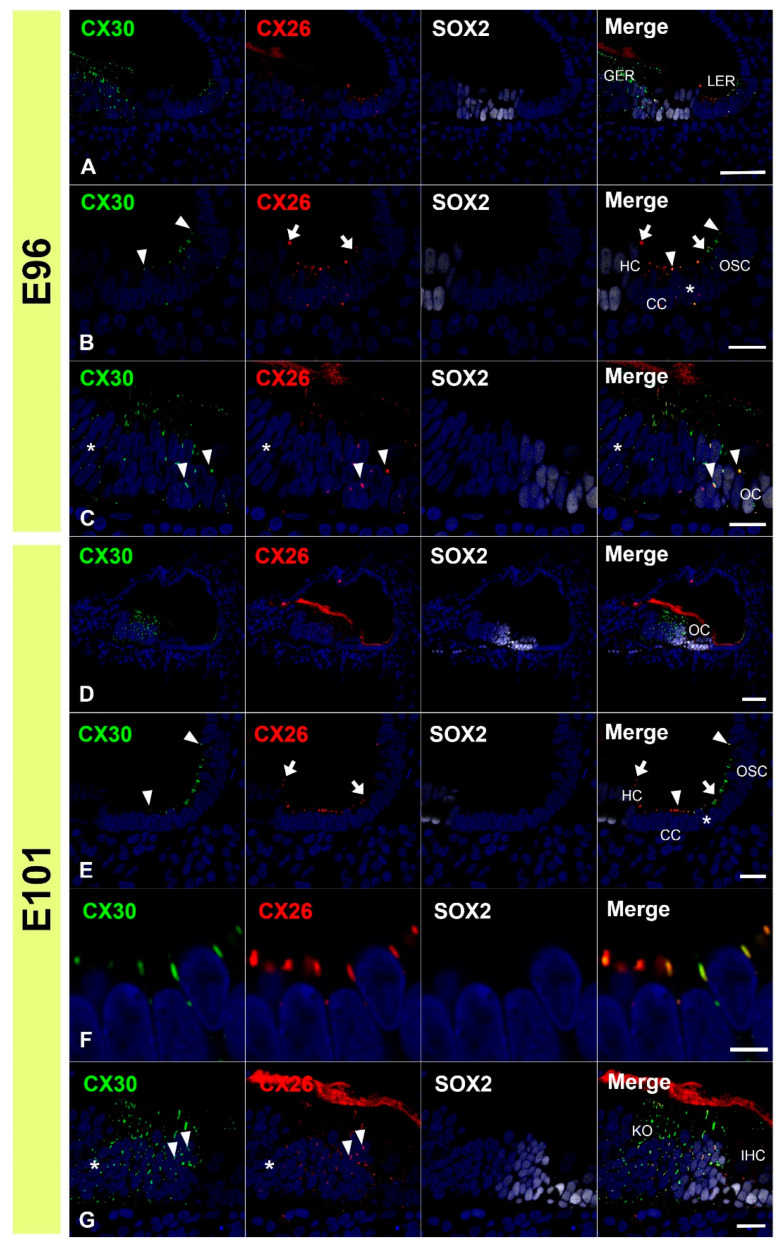
Comparison of the expression patterns of CX26 and CX30 at E96 and E101. (**A**–**C**) At E96, a partial overlap in expression of CX26 and CX30 was observed. In the LER, CX26 expression was detected more on the modiolus side (between arrows in (**B**)), while CX30 expression was detected more laterally (between arrowheads in (**B**)). Overlapping expression was observed in a part of immature Claudius’ and outer sulcus cells (asterisk in (**B**)). In the GER, partial overlap in expression was observed on the lateral side (arrowheads in (**C**)). On the modiolus side, only CX30 expression was detected (asterisk in (**C**)). (**D**–**G**) At E101, partial overlap in the expression of CX26 and CX30 was also observed, as seen at E96. In the LER, CX26 expression was detected more on the modiolus side (between arrows in (**E**)), while CX30 expression was detected more laterally (between arrowheads in (**E**)). Overlaps in expression were observed in some immature Claudius and outer sulcus cells (asterisk in (**E**)). (**F**): A high magnification image of E. In the GER, partial overlap in expression was observed on the lateral side (arrowheads in (**G**)). On the modiolus side, only CX30 expression was detected (asterisk in (**G**)). SOX2 was used to label supporting cells. Nuclei were counterstained with Hoechst stain (blue). Scale bar: 50 µm in (**A**,**D**), 20 µm in (**B**,**C**,**E**,**G**), 5 µm in F. GER: greater epithelial ridge, LER: lesser epithelial ridge, CC: Claudius’ cells, HC: Hensen’s cells, OSC: outer sulcus cells, OC: organ of Corti, KO: Kölliker’s organ, IHC: inner hair cells. (**A**–**F**): basal turn.

**Figure 6:**
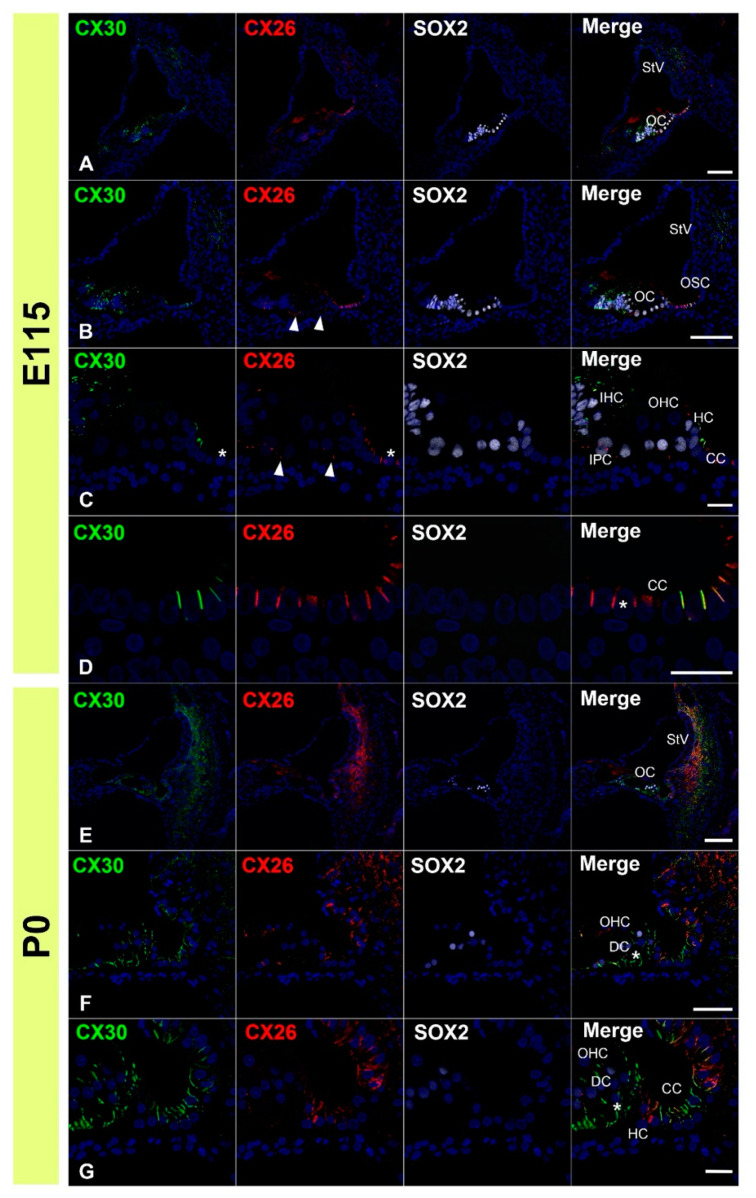
Comparison of the expression patterns of CX26 and CX30 at E115 and P0. (**A**–**D**) At E115, only CX26 expression was detected on the basal side of supporting cells of the organ of Corti (arrowheads in (**B**,**C**)). In Hensen’s cells, both CX26 and CX30 expressions were detected on the cells’ upper side. On the modiolus side of Claudius’ cells, only CX26 expression was detected (asterisks in (**C**,**D**)). (**E**–**G**) At P0, both CX26 and CX30 expressions were detected from the outer sulcus cells to the inner sulcus cells except for the inner and outer hair cells. However, in the organ of Corti, a relatively high expression of CX30 was observed (asterisk in (**G**)). SOX2 was used to label supporting cells. Nuclei were counterstained with Hoechst stain (blue). Scale bar: 100 µm in (**A**,**B**,**E**), 50 µm in (**F**), 20 µm in (**C**,**D**,**G**). StV: stria vascularis, OC: organ of Corti, OSC: outer sulcus cells, IHC: inner hair cells, OHC: outer hair cells, CC: Claudius’ cells, HC: Hensen’s cells, IPC: inner pillar cells, DC: Deiters’ cells. (**A**–**G**): basal turn.

Next, we compared the expression patterns of CX26 and CX30 by co-immunostaining. At E96, partial overlap in the expression of CX26 and CX30 was observed (Figure 5A–C). In the LER, CX26 expression was detected more on the modiolus side, while CX30 expression was detected more laterally. Overlapping expression was observed in some immature Claudius cells and outer sulcus cells. In the GER, partially overlapping expression was observed on the lateral side. On the modiolus side of the GER, however, only CX30 expression was detected. At E101, partial overlap in the expressions of CX26 and CX30 was also observed, as seen at E96 (Figure 5D–G). In the LER, CX26 expression was detected more on the modiolus side, while CX30 expression was detected more laterally. Overlapping expression was still observed in some immature Claudius cells and outer sulcus cells, as observed at E96. In the GER, partially overlapping expression was observed on the lateral side. On the modiolus side, only CX30 expression was detected. At E115 (Figure 6A–D), only CX26 expression was detected on the basal side of the outer pillar cells and Deiters’ cells. In Hensen’s cells, both CX26 and CX30 expressions were detected on the upper side of the cells. On the modiolus side of the Claudius cells, only CX26 expression was detected, while both CX26 and CX30 were detected on the lateral side of Claudius’ cells. At P0, both CX26 and CX30 expression were detected from the outer sulcus cells to the inner sulcus cells, except for the inner and outer hair cells (Figure 6E–G). A relatively high expression level of CX30 was observed in the organ of Corti, while a relatively high expression level of CX26 was detected in Hensen’s cells, Claudius’ cells, and outer sulcus cells.

### 3.4. Comparison of the Expression Patterns of CX26 and CX30 in Lateral Wall Fibrocytes

**Figure 7:**
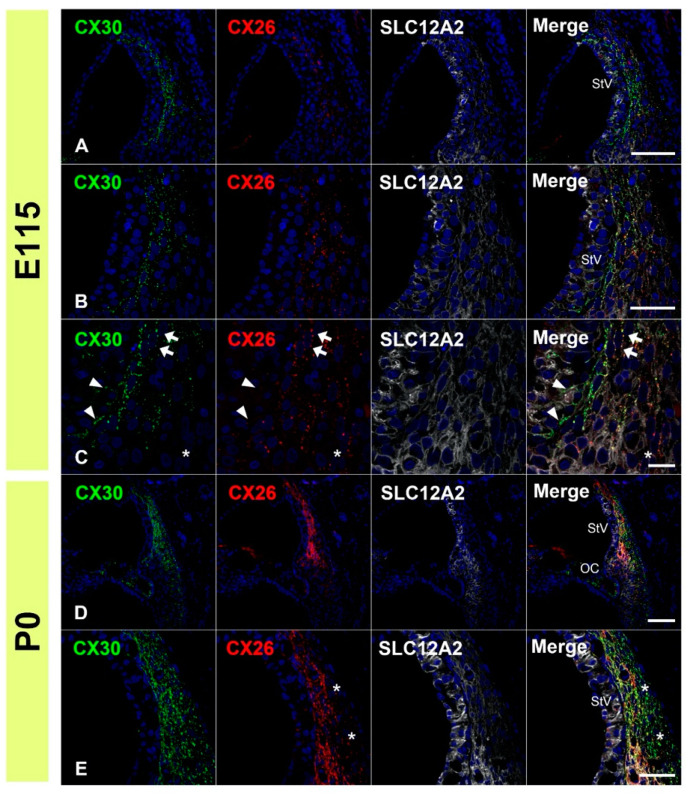
Comparison of the expression patterns of CX26 and CX30 at E115 and P0 in lateral wall fibrocytes. (**A**–**C**) At E115, both CX26 and CX30 expressions were detected in the upper half of the spiral ligament fibrocytes. At this stage, a relatively high expression of CX30 was observed. On the modiolus side (stria vascularis side), CX30 expression was predominantly observed, and, in several cells, only CX30 expression was detected (arrowhead in (**C**)). CX26 expression was observed more laterally, and, on the lateral side of fibrocytes, only CX26 expression was detected (asterisk in (**C**)). In the central region, overlapping expression of CX26 and CX30 was observed (arrow in (**C**)). (**D**–**E**) At P0, both CX26 and CX30 were observed in the lateral wall fibrocytes and lateral side membrane of the basal cells of stria vascularis. In contrast to E115, the expression of CX30 was observed more broadly. On the lateral side of fibrocytes (type III fibrocytes), only CX30 expression was observed (asterisk in (**E**)). SLC12A2 (NKCC1) was used to label stria vascularis and lateral wall fibrocytes. Nuclei were counterstained with Hoechst stain (blue). Scale bar: 100 µm in (**A**,**D**), 50 µm in (**B**,**E**), 20 µm in (**C**). StV: stria vascularis, OC: organ of Corti. (**A**–**E**): basal turn.

Finally, we compared the expression patterns of CX26 and CX30 in lateral wall fibrocytes. Both CX26 and CX30 were detected in these fibrocytes after E115. Therefore, we investigated the cochlea at E115 and P0. At E115, both CX26 and CX30 expression was detected in the upper half of spiral ligament fibrocytes (Figure 7A–C). At this stage, a relatively high expression of CX30 was observed in spiral ligament fibrocytes. On the modiolus side (stria vascularis side), CX30 expression was predominantly observed, and, in several cells, only CX30 expression was detected (arrowhead in Figure 7C). CX26 expression was observed more laterally, and, on the lateral side of fibrocytes, only CX26 expression was detected (asterisk in Figure 7C). In the central region, there was an overlapping expression of CX26 and CX30 (arrowhead in Figure 7C). At P0, both CX26 and CX30 were observed in the lateral wall fibrocytes and lateral side membrane of the basal cells of the stria vascularis. In contrast to E115, the expression of CX30 was observed more broadly. On the lateral side of fibrocytes (type III fibrocytes), only CX30 expression was observed.

## 4. Discussion

**Figure 8:**
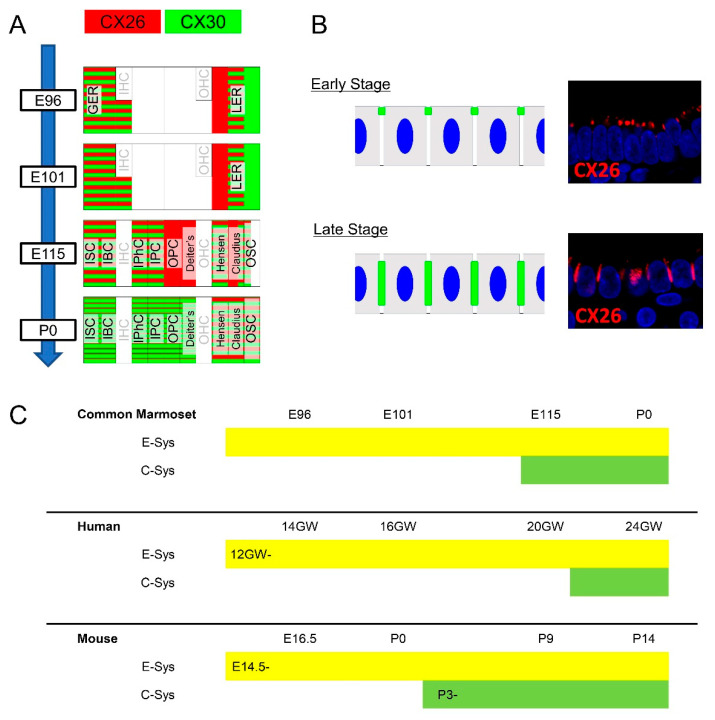
Schema of spatiotemporal expression patterns of CX26 and CX30 (**A**) Schema of the expression pattern of CX26 and CX30 in the organ of Corti. CX26 and CX30 showed their specific expression pattern and were not always coincident with each other. (**B**) Schema of changes in the intracellular localization of connexins. In the early stage, CXs were observed as smaller plaques at the top of lateral cell walls. In contrast, in later stages, its expression was observed as larger plaques between the cells. (**C**) Schematic diagram of expression of CXs comparing common marmoset, human, and mouse. Compared with the mouse, common marmoset and humans have a relatively longer time gap between CXs expressions in E-sys expression and C-sys expression.

In the present study, we observed dynamic spatiotemporal expression changes in the connexins of developing primates. As summarized in Figure 8, CX26 and CX30 expression dynamically changed depending on the developmental stage. These spatiotemporal changes were not limited to cells expressing connexins and were observed in the intracellular localization of connexins. For example, in Claudius’ cells, connexin expression was observed in smaller plaques at the top of lateral cell walls in the early stages (Figure 8B). In contrast, in later stages, its expression was observed as larger plaques.

The distribution of connexins in the cochlea can be divided into two distinct cellular networks: the epithelial gap junction system (E-sys) and the connective tissue system (C-sys) [39]. The organ of Corti is included in the E-sys, while the lateral wall fibrocytes belong to the C-sys. To date, several studies related to connexins in the developing cochlea of rodents were reported, and it is known that expression of connexins in the E-sys is followed by an expression in the C-sys. In previous studies conducted in mice [40], CX26 and CX30 expression in the E-sys was first observed at E14.5. After birth (later than P2), connexin expression was also observed in the C-sys. In the developing rat cochlea, expression of both connexins was observed in E-sys at E17 and in C-sys at postnatal day 3 [18].

Little is known about expression changes in the connexins of the human fetus because of the rarity of well-prepared samples, especially from the late phases of gestation. Most of this scarcity stems from ethical concerns in many countries. A previous investigation of CX26 and CX30 expression patterns using a well-prepared human fetus was reported by Locher et al. in 2015 [41]. This study used relatively early-stage human fetuses from 10.4 GW to 18 GW to observe connexin expression. The authors reported that expression of CX26 and CX30 was significantly increased by 12 GW and was observed on both sides of the developing organ of Corti. At 18 GW, connexin expression was detected in Kölliker’s organ and the cells lining the outer sulcus, including Claudius’ cells and developing root cells. However, in the previous study, connexin expression was observed in neither the organ of Corti nor the spiral ligament fibrocytes of the human fetus at least up to 18 GW. In contrast, their expression was observed in adult humans. In another report, Kammen-Jolly et al. reported CX26 expression in C-sys at 24 GW [42].

In comparison to these previous studies, our observations in the common marmoset were more similar to the human findings than the rodent ones. We did not observe any CX26 or CX30 expression in the organ of Corti or lateral fibrocytes until E101, which is equivalent to 16 GW in human fetuses with cochlear development [35]. We first observed the expression of these connexins in the organ of Corti and C-sys at E115, which is equivalent to 20 GW in human fetuses with cochlear development. These interspecies observations indicate that the E-sys commonly develop before the C-sys. In addition, the expression of connexins in the organ of Corti was observed after expression in the epithelial cells of the organ of Corti. Rodents showed relatively shorter-expression gap times (less than 1 week) between the development of these two systems. In the common marmoset cochlea, however, connexin expression was observed only in the E-sys for approximately 3–4 weeks, followed by C-sys expression. These developmental periods, in which connexins are only expressed in the cochlear epithelium, include several essential steps, including hair cell maturation, synaptic formation, and development of the stria vascularis. Therefore, at least in primates, our results indicate that these developmental steps occur before C-sys formation and are therefore independent of C-sys functions, such as potassium cycling in lateral fibrocytes. A schematic diagram of expression changes of CXs comparing common marmoset, human and mouse was shown in Figure 8C.

In addition, we observed dynamic changes in the intracellular localization of connexin plaques in sensory epithelial cells, which has not been reported previously. These changes were most typically observed in Claudius cells (Figure 8B) but were also observed in other epithelial cells, as well as in Deiters’ and Hensen’s cells. Our observations suggest that other unknown factors control the intracellular localization of connexin expression, which may influence the biochemical properties of connexin channels. It is also possible that other factors recruit connexin proteins, which may lend specific properties (e.g., selectivity or activity) to connexin channels at specific stages. CX26 and CX30 form heteromeric hemichannels, with different selectivities for homomeric versus heteromeric hemichannels [19,43,44]. Additionally, the formation of heterotypic channels provides a greater variety in channel properties, including conductance, permeability, and gating, which cannot be obtained with a single connexin [45]. Our results indicate that this intracellular localization may modify the previously reported functional variety of gap junctions. A future study should be conducted focusing on changes in the intracellular localization and molecular selectivity of connexin channels in the primate cochlea.

The mosaic expression pattern of CX26 and CX30 provides novel insights into the function of these connexins during cochlear development. We observed that CX26 and CX30 showed specific expression patterns, with a partial but not complete overlap in the sensory epithelium and lateral fibrocytes during development. This finding indicates that a lack of one connexin cannot be entirely compensated for by another connexin, at least during some developmental stages in the cochlea of the common marmoset and potentially other primates. For example, at E115, the modiolus half of Claudius’ cells showed only CX26 expression, while the lateral side showed both CX26 and CX30 expression. Therefore, on the modiolus side of Claudius’ cells, CX26 loss cannot be compensated for by CX30 at this particular stage. In addition, Deiters’ cells also expressed only CX26 at E115. The separate expression patterns of CX26 and CX30 in supporting cells were transient and not observed at P0. However, these expression patterns were observed in relatively late stages of cochlear development in the Claudius’ and Deiters’ cells of primates, after hair cell maturation and synapse formation were already proceeding [35]. Functional disturbances in CX26 gap junctions at this stage, which cannot be compensated for by CX30, might cause primate-specific phenotypes. This finding may help to explain potential mechanisms underlying human hearing loss phenotypes related to *GJB2* variants, which were previously unclear based on rodent models. Future functional analyses are awaited.

Our results are also helpful for the development of future therapeutic approaches to diseases related to connexin mutations. Gene therapy is thought to be a feasible therapeutic approach for congenital hearing loss caused by connexin mutations [30,31,32]. While the several progression type hearing loss caused by certain *GJB2* genotypes might be treated by gene therapy effectively still in adults, the timing of gene delivery in utero or after birth is imperative in most patients. In a rodent model, it was reported that deafness induced by a CX26 deficiency is associated with a cochlear developmental disorder and is not determined by EP reduction [14]. Our results indicate that expression of CX26 in the E-sys is more critical for a normal hearing acquisition than C-sys. Therefore, in human patients, modifications to CX26 by gene therapy are required before 16 GW, which is a relatively early phase of gestation. The variety of expressions both in their mosaic patterns and the plaque sizes may also explain variable hearing levels in either *GJB2* or *GJB6* related-hereditary hearing loss. It would be helpful to use primates as a preclinical model before applying these gene therapies to human patients.

Our results indicate that spatiotemporal patterns of connexin expression are complicated, with differences between rodents and primates, suggesting that approaches developed in rodents may not be appropriate in human patients and may require modifications. Our results also emphasize the importance of careful observation of differences in the developmental changes of connexins between humans and rodents.

## 5. Conclusions

We investigated the developing embryonic cochlea of the common marmoset and observed dynamic spatiotemporal expression changes in connexins in this primate’s cochlea. These observations also revealed interspecies differences in connexin expression patterns between previous rodent models and the common marmoset. These primate-specific expression patterns suggest the existence of primate-specific control of connexin expression and primate-specific functions of these gap junction proteins.

Our observations indicate that CX26 and CX30 expressions in marmoset appears to diverge more widely than what was observed in rodents, with clear implications regarding the constitution and properties of connexin channels and suggest that the dynamic expression changes of connexins that were observed in common marmosets may also exist in humans, which has not previously been investigated due to technical and ethical issues. However, our study underscores the importance of future detailed examinations of connexin expression patterns in human fetuses, especially in the late phases of development. Moreover, our results indicate that future therapeutic strategies targeted at genes established in rodent models may not be as effective for human patients. It is crucial to find animal models that more closely resemble the situation in human patients, and common marmoset can now be genetically modified. Therefore, it would be helpful to use primates as a preclinical model before applying gene therapies to human patients.

## Data Availability

Not applicable.
